# Conceptualizing and contextualizing functioning in people with severe mental disorders in rural Ethiopia: a qualitative study

**DOI:** 10.1186/s12888-015-0418-9

**Published:** 2015-03-04

**Authors:** Kassahun Habtamu, Atalay Alem, Charlotte Hanlon

**Affiliations:** School of Psychology, College of Education and Behavioral Studies, Addis Ababa University, P.O. BOX: 1176, Addis Ababa, Ethiopia; PhD students, Department of Psychiatry, School of Medicine, College of Health Sciences, Addis Ababa University, Addis Ababa, Ethiopia; Department of Psychiatry, School of Medicine, College of Health Sciences, Addis Ababa University, Addis Ababa, Ethiopia; Centre for Global Mental Health, Institute of Psychiatry, Psychology and Neuroscience, Kings College London, London, UK

**Keywords:** Disability, Mental disorder, Mental health, Developing countries, Poverty, Stigma

## Abstract

**Background:**

The functional outcome of people with severe mental disorders (SMD) is purported to be better in low- and middle-income countries compared to high-income countries; however, cross-cultural measures of functioning may not capture adequately the relevant functional activities in rural, non-Western settings. This study aimed to gain in-depth understanding of day-to-day functioning in a rural Ethiopian setting and the functional impairments associated with SMD.

**Method:**

A qualitative study was carried out in the Butajira area, south Ethiopia. In-depth interviews were conducted with people with SMD (n = 6), religious healers (n = 2) and psychiatric nurses (n = 2). Four focus group discussions were carried out with caregivers of people with SMD (n = 37) and one with project outreach workers (n = 5). A thematic analysis approach was used.

**Results:**

Participants emphasized that functional impairment in people with SMD arose not only because of the symptoms associated with the illness, but also due to poverty, social exclusion and lack of social support. Within this rural community, the ability to work productively, engage in family life, maintain self-care and fulfill social obligations were the most highly valued domains of functioning. A wide range of farming tasks were elaborated in detail and noted to be of varying levels of difficulty. Although many people with symptomatic SMD were reported to be able to carry out simple farming tasks, this was distinguished from effective farming. Gender differences were most apparent in the domains of work and family life. Impaired functioning was reported to have a critical immediate impact on survival and longer-term impacts on the lifetime opportunities of people with SMD, their caregivers and the younger generation within the family.

**Conclusions:**

The study indicates that tackling social exclusion and poverty is needed alongside medical treatment through contextual community based rehabilitation programs. The gendering of functional roles and the complexity of work activities in this subsistence farming community lend support to arguments for locally contextualized measures of functioning in people with SMD.

## Background

The notion that the clinical and functional outcome of schizophrenia might be better in low- and middle-income countries (LAMICs) when compared to high- income countries has been embraced for the last three decades [1-5]. The evidence to support this contention came largely from a series of multi-country studies conducted by the World Health Organization (WHO) between the late 1960s and the 1980s [6-8]. The better outcome of schizophrenia in the WHO study LAMIC sites was hypothesized to be explained by family support, styles of interaction, favorable attitudes among family members and the community, availability of non-stressful employment settings, higher levels of marriage and supportive work place colleagues. Recently these findings have been challenged [1,4,9]. First, methodological limitations of the WHO studies, including recruitment from treatment centers and high levels of attrition, may have biased the LAMIC studies to better patient outcomes [1-5,9]. Second, recent systematic reviews of studies conducted in LAMICs indicate that the prognosis of people with schizophrenia in LAMICs does not appear to be as favorable as was previously thought [2,5].

Furthermore, many factors known to be associated with poorer prognosis in schizophrenia are more commonly encountered in LAMICs: low levels of awareness about mental illness, delays in seeking treatment and consequent prolonged duration of untreated psychosis, lack of treatment availability, over-burdened families [9]; stigma, discrimination and abuses, high mortality rates and suicide [2,10,11]; high levels of poverty, income inequality, and, in some settings, substantial social disintegration [4]. The lack of evidence to support any beneficial effect of sociocultural factors upon schizophrenia prognosis in LAMICs has also been highlighted [3]. Lastly, it has been argued that functional recovery is a complex and multi-dimensional concept and that meaningful comparison across cultural settings may not be achievable with instruments developed in Western and high-income country settings [12]. In view of this, investigators have called for in-depth qualitative exploration of the concepts of functioning, disability and functional recovery in people with schizophrenia in LAMIC settings [2-4].

There is also a dearth of evidence on the functional outcomes of severe mental disorders aside from schizophrenia, including bipolar disorder and depression with psychotic features, in LAMICs. In the available literature, there is little evidence of a favorable outcome in these conditions either. In a course and outcome study of bipolar disorder in rural Ethiopia, the level of functioning was significantly lower than general population norms, both at baseline and follow up [13]. A study of the functional outcome of major depression in rural Ethiopia [14] showed that disability scores were significantly higher in people with recovered depression than in those with no depression.

The concept of functioning includes aspects such as the capacity to work, study, live independently, and engage in recreation and romantic life [15-18]. However, the domains of functioning differ in different cultures as people are expected to have different roles in those cultures [19]. This is particularly the case for rural low-income country settings, compared to more Western-exposed urban settings. Understanding of the concept of impaired functioning has changed from biomedical and social perspectives to the biopsychosocial model in the last decades [20], emphasizing the dynamic and bidirectional interrelationships between health conditions and contextual factors (personal and environmental) [21]. The WHO developed the International Classification of Functioning, Disability and Health (ICF) [18,21] as a universally accepted conceptual framework to understand and classify impaired functioning. In the ICF, impaired functioning is defined as *“a difficulty in functioning at the body, person or societal levels, in one or more life domains, as experienced by an individual with health condition in interaction with contextual factors”* [22]. However, having a universally accepted framework to understand functioning is challenging due to the contextual nature of the concept, which brings a serious problem to the use of exiting cross-cultural measures of functioning in LAMICs.

The primary aim of the current study was to gain in-depth understanding of functioning and disability in people with SMD in rural Ethiopia in order to inform the future development of a socio-culturally appropriate measure of functional status. The study sought to situate functioning in people with SMD within their general life experience and within the norms and expectations of the wider community. Moreover, the study was intended to identify key tasks that an adult person in the rural Ethiopian context is expected to accomplish with a view to evaluating the adequacy of existing scales and operationalizing new scale items to measure functional impairment.

## Methods

### Study setting and context

People in rural Ethiopia are dependent on agriculture for their main livelihood. The majority of the people rely on subsistence farming and have to work to survive. However, the majority of working age adults have their own land to farm or they can be employed by other farmers who have land and thus have the opportunity to work. There is no state welfare system in Ethiopia. The present study was conducted in and around the town of Butajira, which is located 135 km south of Addis Ababa, the capital city of Ethiopia. The Butajira area is found within the Gurage administrative zone, in the Southern Nations, Nationalities and Peoples’ Region. There has been a demographic surveillance site (DSS) in the area since 1987 [23], which provides the necessary infrastructure and support for conduct of community-based research studies.

There are both urban and rural dwellers in the Butajira area, but the majority of the people live rurally. The topography is variable, ranging from hot, dry lowlands to cool mountainous areas [24]. The main livelihood of the people in the rural part of the area is farming, whereas small scale trading is common in the town [25]. While maize is the main subsistence grain, khat and chilli pepper are the main cash crops [24]. The majority of the people in the Butajira area are Muslims or Orthodox Christians, and most are engaged in activities related to their religious beliefs. Administratively, the area is divided into *Woredas* (districts), and the population in each district is living in different *Kebeles* (sub-districts). Different informal social structures, such as *Idir* (funeral insurance groups) and *Iqub* (local saving groups) are very common in the area.

Both traditional and modern methods of treatment are used by the people in the Butajira area for their mental health problems. Holy water is the most common traditional treatment for people with SMD. Holy water is spring water that is regarded as holy, given by God or by one of the saints, for healing the sick. Healing comes by baptism or drinking the water or both. The ceremony is usually associated with prayer and the reading of the holy books by priests. Biomedical mental health care services are limited to the out-patient psychiatric unit of Butajira Hospital, which is led by two psychiatric nurses. For more than 12 years, the Butajira area has hosted a population-based cohort study of over 900 people with SMD investigating the course and outcome of SMD in this setting [25].

### Study design and sample

A qualitative study was carried out involving a range of informants expected to have firsthand experience of the functioning of people with SMD in this rural Ethiopian setting: people with SMD, caregivers, health care workers, project outreach workers and religious healers. Ten one-to-one in-depth interviews and five focus group discussions (FGD) were carried out. Six in-depth interviews were conducted with people with SMD who were selected purposively from the Butajira SMD cohort. Interviews were used for people with SMD as there were concerns that groups could be stressful and too confronting. Project outreach workers were asked to identify people with SMD who were currently well and able to express themselves. Two psychiatric nurses, who have been working in the Butajira Hospital Psychiatric Unit for some years, were also interviewed.

As the majority of people with SMD are taken to Holy Water healing sites within the Orthodox Christian church, we also interviewed two Orthodox Christian healers from Butajira town. We were not able to use FGDs for these participants as it was difficult to bring together healers from different churches.

Four FGDs were carried out (six to ten participants in each group) with caregivers of people with SMD in the Butajira cohort. The selected caregivers were all close family members (parents, sons/daughters, siblings or husband/wife) who were living with the person with SMD and balanced in terms of gender mix. One FGD was conducted with all of the Butajira SMD cohort study project outreach workers, who were five in number (two females and four males). FGD was used with caregivers and project outreach workers to elicit more information through their interaction and to encourage sharing of their experiences. Socio-demographic characteristics of the interview and FGD participants are presented in Table 1.

**Table 1:** **Socio-demographic characteristics of participants**

	**Interview**	**Interview**	**Interview**	**FGD**	**FGD**
	**(People with severe mental illness)**	**(Psychiatric nurses)**	**(Religious healers)**	**(Caregivers)**	**(Project workers)**
**Number of participants**	6	2	2	37	5
**Age categories (years)**					
**<25**	1	0	0	3	0
**25-34**	0	0	1	9	3
**35-44**	2	1	1	11	2
**45-59**	3	1	0	9	0
**60 and above**	0	0	0	5	0
**Gender**					
**Male**	3	2	2	21	3
**Female**	3	0	0	16	2
**Residence**					
**Rural**	5	0	0	31	0
**Urban**	1	2	2	6	5
**Marital status**					
**Single**	1	1	1	5	0
**Married**	3	1	1	24	5
**Widowed**	0	0	0	6	0
**Separated**	2	0	0	2	0
**Education**					
**Can’t read and write**	2	0	0	11	0
^**1**^ **Read and write only**	2	0	2	11	0
**Primary**	1	0	0	10	0
**Secondary**	0	0	0	3	2
**Post-secondary**	1	2	0	2	3
**Religion**					
**Muslim**	3	0	0	17	1
**Orthodox**	3	2	2	18	4
**Protestant**	0	0	0	2	0
**Occupation**					
**Farming**	4	0	0	22	0
**Trading**	2	0	0	12	0
^**2**^ **Employed**	0	2	2	3	5

^1^“Read and write only” means those who have no formal education but they have basic literacy and they can read and write. There are different ways of getting literacy education in Ethiopia, such as adult literacy program.

^2^“Employed” means a person has a monthly salary being employed in a private, government, religious or other non-governmental institution.

### Data collection procedures

All in-depth interviews and FGDs were conducted in Amharic, the official language of Ethiopia. The first author (KH), who has training and experience in qualitative data collection, conducted all FGDs and in-depth interviews. While KH was moderating FGDs, a note taker summarized the discussions and noted the non-verbal communication. The other co-authors (CH, AA), who are senior psychiatrists and mental health researchers in Ethiopia, attended four in-depth interviews with patients and one FGD with caregivers.

The in-depth interviews with patients and psychiatric nurses and the FGDs with caregivers were conducted at Butajira Hospital Psychiatric Unit, whereas in-depth interviews with religious healers were held in their respective churches at the site of Holy Water treatment. The FGD with project outreach workers was conducted at the Department of Psychiatry, Addis Ababa University guesthouse in Butajira. Privacy was assured at all times. All participants, except the psychiatric nurses and project outreach workers, received modest remuneration for their time and transportation costs. All interviews and FGDs were tape-recorded, with the consent of the participants. Generally, in-depth interviews lasted between 22 to 70 minutes, whereas FGDs lasted between 65 minutes and 81 minutes.

Participants were asked about the experiences, symptoms and day-to-day activities of people with SMD focusing, in particular, on tasks that people with SMD are, and are not, able to do during illness onset, recovery and relapse. Planned probes, which included questions related to disability in terms of understanding/concentration, interpersonal relationship, self-care, work, social participation and mobility, were identified from the WHO’s Disability Assessment Schedule (WHODAS 2.0) which is based on the ICF [26,27].

### Data analysis

The in-depth interviews and FGDs were transcribed verbatim in Amharic by an experienced transcriber. The first author translated all transcripts into English, and one of the second authors (CH) read and gave feedback on all of the translations. Open Code 4.02 computer software [28] was used to facilitate data management.

Data analysis was undertaken in parallel with data collection, with frequent discussion of the emerging themes and issues between the first and last author. Thematic analysis [29] was used to identify the prominent issues from the data. Themes and categories were compared between men and women and between the different respondent groups. The first and last authors coded two transcripts independently, and coding schemes were compared and disagreements were discussed and consensus reached. The first author coded all the remaining transcripts applying the already identified codes and drawing upon additional codes where the data required, frequently discussing with the last author. Higher order codes were derived from the primary codes with thorough discussion between the first and last authors. Similarly, overarching themes were developed from the higher order codes. Illustrative quotes were selected for each theme.

### Ethical considerations

Ethical approval was obtained from the College of Health Sciences Institutional Review Board, Addis Ababa University. Informed consent was sought and recorded in writing prior to participation in the study. For non-literate participants, witnessed consent was sought and recorded. That is, the information sheet was read to the participant and he/she gave finger print while another literate person witnessed with signature that the information sheet is fully described to the participant by the researcher.

## Results

**Figure 1:**
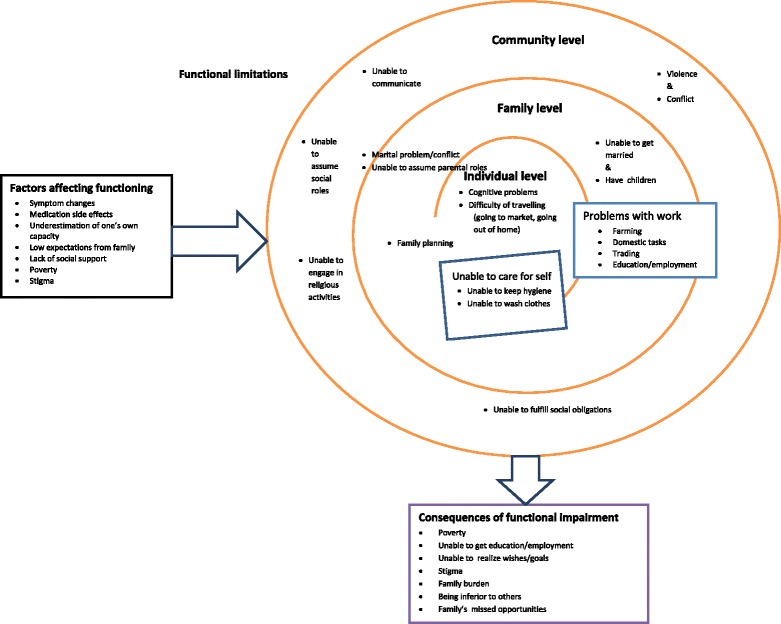
**Conceptual model of the functioning of people with SMD.**

Gender patterning of functioning and impairment was evident and there were differences of emphasis between the three categories of respondents (people with SMD, caregivers and professionals). Although mentioned by a few participants, differences in functional roles across the different religious groups and the importance of religious roles for social functioning were not emphasized. Participants rather focused on those functional tasks that were crucial for their survival. Functioning in people with SMD was found to be conceptualized in terms of three interrelated themes: (1) the broader context influencing functional impairment; (2) activities required to live and survive in rural Ethiopia; and (3) consequences of functional impairment. Based on the findings, a conceptual model was developed (see Figure 1 below), which illustrates how the three themes were related to one another.

1. The broader context influencing functional impairment.

In describing the context of SMD and functioning, participants identified factors that impeded or facilitated the day to day activities of people with SMD in the setting. Although symptoms of mental illness were mentioned by all participants as a key factor affecting performance, in all cases the negative impact of a range of non-symptom-related factors was also emphasized: at the level of the person with SMD (underestimating one’s own capacity and the impact of medication side effects), from family members or caregivers (a lack of support, reluctance to give the person with SMD autonomy or freedom, lowered expectations about the capabilities of the person with SMD, exaggerated fear which might lead to unnecessary restraint) and the wider social and economic conditions (poverty, stigma and discrimination).

### Patient related factors

The majority of participants emphasized that people with SMD would be in a better position to accomplish their day to day tasks when the severity of symptoms was reduced or when symptoms had disappeared altogether. One of the project outreach workers described how illness symptoms affected the functioning of people with SMD as follows.

*When we sit and talk together, I asked one patient the question “why don’t you work?” I said to him “if you do farming, you can be a rich person”. Then, he told me that ‘when I am farming, I see something and it tells me to stop doing this and then I just sit down’. At another time, I happened to tell a patient that if you work hard, you can buy clothes, and you can also support others. When I said this to him, he told me that he is the government of the country. He said that he is governing the country and so “why do I need to work?” (Project outreach worker, female, 28, urban)*

One of the psychiatric nurses also spoke about the effect of illness symptoms on functioning as follows.

*Symptoms, specially the severe symptoms make them [people with SMD] lose energy. For instance, if it is depression, their body loses energy. They lose their energy, get very tired, and “their body will be like cloth” [an Amharic idiom]. So, they will not be able to wash their clothes, prepare their food, move from one place to another, and go to the market and buy the thing they want to buy.(Psychiatric nurse, male, 45, urban)*

One of the important factors to impede the functioning of people with SMD was said to be underestimation of one’s own capacity on the part of the people with SMD. Considering mental illness to be a non-curable, lifelong problem, and influenced by social pressures such as stigma and discrimination, respondents with SMD expressed the view that they would not be able to work and be successful in life. Under-estimation of one’s own capacity in people with SMD was due to the loss of their self-efficacy and self-esteem as a consequence of the attitudes and beliefs described above. People with SMD also reported that side effects of psychotropic medications, such as loss of energy and fatigue, impeded their ability to engage in their daily activities.

### Caregiver-related factors

All groups of respondents reported that family members or caregivers played a pivotal role in improving or worsening the functioning of people with SMD. Family members could improve the functioning of people with SMD that they are caring for through social support; and could further impair functioning by denying the autonomy of the person with SMD and having low expectations about the capacity of people with SMD to function. One of the psychiatric nurses described this as: *“….at home also family members prevent them [people with mental illnesses] from doing things when they try to engage themselves in some tasks.”* Similar attitudes were said to prevail in the community at large. One of the religious healers commented as follows:

*The community itself doesn’t allow them [mentally ill people] to work. The community considers them as ill and rather than encouraging them to work, people from the community advise them to go to holy water places or to hospital. People from the community comment to them “you are ill and how can you work?”*

*(Religious healer, male, 35, urban)*

### Social exclusion and economic conditions

Social and economic conditions were reported by all groups of participants to be important factors affecting the functioning of people with SMD. Stigma associated with mental illness was considered to deprive people with SMD of social participation, interpersonal relationships, marital and family life, and even from employment.

*They [people with mental illnesses] are considered as inferior in the community and if they want to get married, people in the community will rumor that they are mentally ill and parents will not allow giving their daughters/sons to this kind of persons. So, the community doesn’t have a positive attitude towards people with mental disorders. The community considers people with mental disorders as inferior.*

*(Project outreach worker, male, 36, urban)*

*When there are community meetings in the Kebele, the Chair of the Kebele doesn’t want me to be there. People say that he [the patient] doesn’t act normal in meetings.(Man with SMD, 45, rural)*

Poverty was found to have both direct and indirect effects on the functional impairment of people with SMD. Unable to fulfill one’s own and family member’s needs, such as needs for food and clothing, could be an immediate factor to illness relapse and consequently to disability. Participants reported that people with SMD were likely to be found at the lower economic level in the community, and this would prevent them from getting married and participating in different activities of the community, such as “*Idir*” (funeral insurance groups), *kebele* (sub-district) meetings and religious celebrations. In this regard, a family member commented in the FGD that:

*His [the person with SMD] major problem is related to money and poverty; otherwise he is fine. If he is able to get money, he can work and go well with his family members and his neighbors.(Caregiver, Male, 35, rural, brother of a patient)*

2. Living and surviving in rural Ethiopia: required functional tasks

Participants reported many activities or tasks that people with SMD struggled to accomplish. These tasks were considered to be vital for each and every member of the community to carry out and crucial for one’s own and his/her family’s survival. The most valued activities were related to caring for oneself, working and productivity, assuming family responsibilities, interacting with family members, neighbors and members of the community, engaging in community activities and societal responsibilities.

### Self-care

One of the most serious functional limitations in people with SMD was reported to be problems with self-care. All groups of participants, especially caregivers and project outreach workers, reported that people with SMD had difficulties taking care of themselves, for example, unable to wash themselves and their clothes independently. In line with this, a caregiver commented in the FGD:

*He doesn’t take care of himself unless we tell him to wash or change his clothes. He doesn’t even ask us to wash his clothes or to give him water for washing his body. He doesn’t wash his body or his clothes by his own initiative. You know…it is the children and I who wash his clothes and tell him to wash his body and keep his hygiene.(Caregiver, male, 40, rural, brother of a man with SMD)*

For caregivers, this particular functional impairment increased the burden of caring and was also a potent source of stigma. For people with SMD, self-care was clearly a valued activity that was required in order to be functional within a society. Project outreach workers also reported that they had observed people with SMD being unclean, wearing dirty clothes, and having long finger nails.

### Work

Participants gave strong emphasis to mental illness impairing ability to work. Some of the activities impaired by mental ill-health were limited to men or women and others were expected to be undertaken by both men and women. The most important type of work for men in the study area was reported to be farming, which included seasonal tasks of varying difficulty: ploughing, digging, sowing, weeding, and harvesting. Men were also reported to be expected to carry out small-scale business or trading. Women were expected to do some, but not all, aspects of farming, including growing food in the immediate locality of the home, weeding, and supporting men with ploughing and harvesting. However, the most critical work activities carried out by women were reported to be various domestic tasks: for example, cooking food, cleaning the house, washing clothes, and fetching water. Women were also expected to engage in small-scale business or trading. All groups of participants reported that people with SMD had difficulty to accomplish these tasks with differences during onset, recovery and relapse and also between men and women.

*…farming, animal rearing, bee farming, planting and taking care of trees, farming maize, farming pepper etc. These are the major tasks in this community. But, when we see persons with mental illnesses, they don’t do these tasks very well. Rather they move around with no purpose.(Religious healer, male, 33, urban)*

Respondents emphasized that it was not just a matter of going to work in the fields, but that the quality of the work and productivity had to be taken into consideration. People with SMD themselves acknowledged that they had difficulty to accomplish tasks that were crucial to their survival even when they were in the state of remission.

*If I were healthy, I could have done small business. I do have a donkey at home, and I could have bought grain from Enseno and sold it at Butajira. I could have done trading with the donkey I have at home…so that I could have bought clothes for my children and we could have eaten enough.(Woman with SMD, 49, rural)*

### Inter-personal relationship

Almost all respondents described that people with SMD have problems in their relationships with other people. They were reported to have poor communication with their family members, neighbors and with members of the community at large. Caregivers described a number of situations where the people they care for entered into conflict with others.

*In my case, the problem that I consider as most difficult is she [the person with SMD] easily gets upset. She enters into conflict with the children and other people easily. She misinterprets what others are talking and feels angry.(Caregiver, male, 50, rural, father of a woman with SMD)*

*He [the patient] talks alone; he has little communication and relationship with other people. He doesn’t want to enjoy with other people; he wants to be alone. He stays at home for twenty four hours and doesn’t do anything.(Caregiver, female, 30, rural, wife of a man with SMD)*

However, there were differences in terms of gender and whether the patient was in recovery or relapse. Participants described that patients had very poor communication, tended to instigate conflict with family members, neighbors and with the community, and were more violent against other people when they were in relapse or when their illness was just starting. Compared to women, men with SMD were described as more violent and involved in more conflict and more likely to have very poor communication.

### Community participation

Inability to participate or having little participation in different community activities, such as weddings, funeral ceremonies, holiday celebrations, visiting persons who are sick and postnatal women, and attending local administrative meetings, was another important functional limitation observed in people with SMD. This aspect of functioning was reported to be mandatory for adults in the rural Ethiopian setting if a person was said to be healthy and living a full life. Any adult member of the community was expected to form his/her own family and participate in various community activities; otherwise he/she would be stigmatized and discriminated against and would not be able to receive reciprocal benefits from the community.

People with SMD also acknowledged their limited participation in different community activities due to the illness. Some of them reported that the over-riding reason for their limited social participation was due to being stigmatized and discriminated against. Some of the caregivers indicated that people with SMD that they cared for were able to work, manage the family and assume responsibilities in the family when they were in the state of recovery, but that they still might not be able to go out and engage in different community activities.

*My brother [the person with SMD] is able to work and manage his money. But, he has limited social life. He is careless in terms of his relationship with other people. He even considers social activities and participation in community activities as useless. This is his major problem.(Caregiver, male, 55, rural, brother of a man with SMD)*

### Family life

People with SMD were reported to have problems with regard to family life, particularly with getting married and having children. Respondents reported that negative attitudes from community members were an important reason impeding marriage of a person with mental illness. The other reported reason for reluctance to marry a person with mental illness was due to their poverty. People with SMD were seen to be on the lowest rung of the economic ladder, compared to the general population, due to their difficulties in working productively. The burden associated with caring for a mentally ill person was also considered to be a factor putting people off marrying a person with mental illness. Indeed, if a person with SMD was somehow able to get married, usually due to the support of family and relatives, the study respondents considered that divorce would be almost inevitable.

*He [the patient] was well and started working. By chance, after he got married, his wife left him and went to her parents saying that I don’t want him, he is mentally ill, and I got married to him without being aware that he is mentally ill. Now, I know that he is mentally ill, and I don’t want to live with him. Immediately after she left him and went to her parents, his illness relapsed and now he is spending the day at home chained up.(Caregiver, female, 60, rural, mother of a man with SMD)*

Almost all groups of respondents reported a difference between men and women with SMD in terms of the opportunity they had to get married and have children. Generally, men with SMD were able to make use of the traditional arranged marriage system in rural Ethiopian communities. That is, the relatives of the man with SMD could find a woman and ask her parents to marry the man. However, this kind of arrangement was not possible for women.

*People don’t expect that a women patient would be well in the future. Even after she gets better, she can’t get married. But, the man will easily get married if he is better for six months, even for three months. The man can go to other places and get married.*

*(Project outreach worker, female, 29, urban)*

3. Consequences of functional impairment

The inability of people with SMD to accomplish their daily activities was reported to have a number of devastating consequences, both for people with SMD and their family members. Of these the most serious ones included poverty, inability to get education and employment, difficulty realizing wishes and goals, stigma, a feeling of being inferior to others, family burden and missed opportunities for family members.

### Poverty

Poverty was reported to be the most important consequence of functional impairment in people with SMD; and this was well articulated by a psychiatric nurse as *“there is poverty in everybody’s house here, but they [people with SMD] are living below poverty; you see them sleeping in the street.”* If someone was not able to work, relate well with other people and participate in different community activities, poverty was considered to be inevitable. The impact of functional impairment on economic status was reported to be especially severe when the person with SMD was the household head and breadwinner of the family.

*The problem I have at home is that I can’t buy clothes and change as I need. I can’t buy clothes for my children; they can’t eat whatever they need. There are times when they can’t have dinner. There are times when they spend the night only drinking water. If I were able to work, all these things could have never happened.(Man with SMD, 48, rural)*

It was clearly observed in this study that poverty is both a cause and a consequence to functional impairment and disability. People with SMD, who are in the lower socio-economic status, are likely to have poor functioning, compared to those who are in the upper socio-economic status. Those who are better off had more access to resources, both material and social, that would help them to compensate for their loss of functioning. On the other hand, people who are not able to work are likely to be poor.

Functional impairment was also reported to have an impact on the educational attainment and employment opportunities of people with SMD as well as their family members. Many people with SMD discontinued their education since they were not able to function well in school, due to stigma and because of the symptoms associated with the illness. As a result their employment choices were highly restricted, especially in urban areas.

### Stigma and family burden

Other consequences of functional impairment were reported to be stigma and family burden. People with SMD were stigmatized not only because they had the illness or due to the symptoms associated with the illness, but also because they were not able to care for themselves, engage in productive activities, because they entered into conflict with other people, and because they did not participate in different community activities. Family members reported a heavy burden in caring for people with SMD, related to their social, economic and family life. Family members also discontinued their education due to the economic, social and time burden associated with caring for their mentally ill member. The daughter of a mentally ill woman had the following to say about the burden that her mother’s illness brought to the family.

*I was a student but I dropped out from grade ten, after I promoted to grade eleven just to care for her [the person with SMD]. I decided to discontinue my education and take care of her. I don’t have any other business than taking care of her.(Caregiver, female, 27, rural, daughter of a woman with SMD)*

### Social status and failure to fulfill aspirations

Almost all groups of participants highlighted that people with SMD were considered to be inferior to other people of the same age in their community. This was true with respect to every area of life, such as in terms of their economic wellbeing, status in the community, educational attainment, and success in their marital and familial life. Their role, status, voice and position in the community was very low compared to people of the same age.

*It is this illness which is making them [people with mental illnesses] lag behind other people and not to work hard and get money. Of course, if somebody is mentally ill, it is likely that he/she will be inferior or lag behind his/her friends.(Caregiver, male, 50, rural, father of a woman with SMD)*

Connected to this, respondents also spoke of the fact that people with SMD were not able to fulfill their wishes and goals. A caregiver described this in the FGD as:

*Generally, people with mental illnesses can’t fulfill their wishes and they are inferior to anybody else; they are failures in their life.(Caregiver, female, 60, rural, mother of a man with SMD)*

## Discussion

In this qualitative study, we explored the context and conceptualization of the functioning of people with SMD in a low-income country setting. We found that functional impairment in people with SMD is perceived to be the result of not only symptoms associated with the illness, but also a multitude of factors which can be grouped into patient-related factors, caregiver-related factors, social exclusion and economic conditions. Participants reported that there were a number of different kinds of tasks that people with SMD were not able to accomplish. These tasks are highly valued by family members, neighbors, and the community at large, and they are crucial for one’s own survival and the survival of family members. Furthermore, study participants reported that functional limitations in people with SMD bring about adverse consequences to both people with SMD and their family members, including poverty, stigma, burden, diminished education and employment opportunities, being unable to realize wishes and goals and a sense of being inferior to others.

There is generally limited literature in relation to in-depth exploration of functioning in people with SMD in LAMICs. In qualitative studies from India, Nigeria and Ghana, respondents spoke about the impact of severe mental illnesses on work or occupational functioning, social functioning and daily activities [30,31]; marital prospects [32]; sex life, ability to concentrate and energy for everyday life [33]. On the part of family members, severe mental illnesses were reported to bring about burden and a sense of moral or social failure [34]. Therefore, the present study is generally consistent with qualitative studies conducted in other LAMICs.

Previous quantitative studies conducted in rural Ethiopia investigated the association between socio-demographic and clinical characteristics and functional impairment in people with schizophrenia and bipolar disorder. In a course and outcome study of people with bipolar disorder in rural Ethiopia [13], male sex, rural residence, being married and having a history of treatment with antipsychotic medication at baseline were significantly associated with better functional outcome. The study further showed that severity of depression and mania symptoms was associated with poor functional outcome. In the same setting, symptom scores of people with schizophrenia were inversely associated with improvements in physical and social functioning and role limitations due to mental health problems [35]. Findings in the present study are consistent with these epidemiological studies and may be useful to clarify and contextualize how socio-demographic and clinical characteristics are associated with the functioning of people with SMD.

From high -income countries, there are several previous population-based and clinic-based studies that have investigated factors associated with functional impairment in people with SMD. These include socio-demographic (male sex, older age, lower level of education, urban residence, being unemployed, and lower socio-economic status) and illness characteristics (greater number of episodes, longer duration of illness, younger age at onset of the illness, greater number of hospitalizations, suicide attempts, family history of the illness, history of psychotic symptoms and comorbidity) [16,36], cognitive impairment [37], trauma in childhood [38], substance use [39] and lower level of premorbid functional status [36]. In our study, respondents made little mention of predisposing vulnerabilities in explaining functional impairment (childhood trauma, pre-morbid functional status and cognitive impairment).

From the perspectives of people with SMD, caregivers, and health care providers in this study, clinical features of mental illness did not bring any positive functional benefit within society; for example, mental illness did not open up new occupational roles as healers, as has been believed to be the case in some African cultures. On the contrary, a bleak picture of critical impairment of functions required for survival of people with SMD and the family was conveyed. This study identified a number of factors (including stigma, family burden, human rights abuses, mistreatment and violence by caregivers, caregivers’ underestimation of the patient’s ability to function, severe poverty, lack of autonomy, lack of social support and substance use), which are all threats to the ability of people with SMD to accomplish their day to day tasks. This indicates that functional impairment in people with SMD is the result of not only symptoms associated with the illness, but also other social, economic and family related causes.

The study participants indicated that people with SMD have functional limitations across various aspects of life: self-care, family life, work, interpersonal relationships and participation in community activities. It seems that the domains of functional limitations identified in this study accord with those that are recognized cross-culturally and included within the WHODAS [40]. However, the specific activities/tasks identified under each domain of functioning are less generalizable; they are relevant to the local situation in rural Ethiopia for the survival of both the patient and family members; and are different for men and women. This is consistent with what Bolton and Tang [41] have commented saying that functional tasks vary greatly according to sex, culture and environment.

Epidemiological studies conducted in both high- income and LAMICs showed that people with SMD have functional limitations in different areas of life. Using World Mental Health Survey data, Ormel and colleagues [42] found higher disability ratings for mental disorders, in both high- income and LAMICs, compared to physical disorders. There is ample evidence that schizophrenia is associated with severe and enduring psychosocial deficits [43]. It is believed that decline in social functioning represents an area independent of positive and negative symptoms of schizophrenia [44]. A follow-up study of three cohorts of 3,307 persons with schizophrenia conducted in Finland [43] showed that impairment in social functioning was present in more than 80% of the patients at baseline.

Studies in LAMICs also confirmed that people with SMD have disabilities in different domains of life. Assessment of functioning of people with major depressive disorder, using a locally developed instrument, in Uganda and Rwanda [45,46] showed that functional impairment is significantly associated with severity of symptoms. A follow-up study of people with bipolar disorder in rural Ethiopia [13] found that at baseline, the mean scores of all the domains of functioning of cases were significantly lower than the normative group. Domains of social functioning and role limitations due to mental health problems were significantly lower at follow-up in cases. A study of people with schizophrenia in the same setting [35] showed that the mean scores of all the domains of functioning of the cases were significantly lower compared to the mean scores of the general population, at both baseline and follow-up. A study of a similar nature and in the same setting on people with major depressive disorder [14] indicated that disability scores, as measured by WHODAS-2.0, in all of the six domains were significantly greater for those with persistent depression compared to those who were in complete recovery.

Epidemiological studies (both from high- income countries and LAMICs) showed that people with SMD have functional limitations far below the general population. However, these studies do not indicate the specific and valued functional activities that these people are unable to accomplish. Our study may contribute with regard to this issue by providing contextual detail from a typical low income country setting.

The other important finding of this study is with respect to the impact that impaired functioning may have on both people with SMD and their family members. All participants of this study stressed that functional impairment in people with SMD is associated with stigma, severe poverty, and family burden. In many studies, family caregivers of people with SMD are found to suffer from significant stresses and high levels of burden in terms of cost, time, stigma and missed opportunities [34,47,48]. Hence, our findings, in this regard, are consistent with the literature both from high- income countries and LAMICs.

Our findings indicate that treating illness symptoms alone may not be enough to improve the lives of people with SMD in this setting. A study of the effect of a community based rehabilitation program for people with psychotic disorders in a very-low-resource setting found evidence of effectiveness [49]. A recent meta-analysis of the effect of psychosocial interventions on social functioning in LAMICs found that psychosocial interventions are effective for people with depression [50]. Utilizing locally available resources from the community [51], such as social institutions, churches/mosques and micro-finance institutions and peer and group education may help people with SMD and their family members to maintain social functioning and tackle the poverty resulting from functional impairment. This study also shows that family caregivers not only have significant impact on the impairment and recovery of people with SMD, but are also affected by the care burden of their mentally ill family member. Therefore, involving caregivers in the treatment of people with SMD is indispensable. In addition, socio-culturally appropriate family level interventions should be designed and provided. There is evidence from LAMICs that family interventions improve the functioning of people with SMD [52-54].

A strength of our qualitative study is that it is embedded in a longitudinal study that aims to develop and validate a local functioning scale for people with SMD. Our study was delimited to the rural area of Butajira; which is a typical rural Ethiopian setting and the findings can be generalizable to other areas of rural Ethiopia. However, aspects of the study findings, such as those related to required functional tasks, may not be well generalized to urban areas. This is because functional expectations and ways of life are different in urban and rural areas. Ethiopia, as a country, has a diverse population with over 80 ethnic groups and this would potentially limit the generalizability of the findings to other rural parts of Ethiopia. Nevertheless, social systems and main livelihoods, which are important determinants of understanding functioning, are very similar in the majority of the Ethiopian population.

Another possible limitation of this study would be our failure to include other groups of participants such as health extension workers, community leaders, elders and traditional healers, which might have additional perspectives to the context of functioning and SMD. We do believe, however, that those groups who were included in the study are very familiar and close to people with SMD and including these other groups couldn’t significantly change our conclusions. Other potential limitation of the study would be the sampling strategy we used. We purposively selected people with SMD who were well and able to express themselves. We also selected caregivers who were able to speak Amharic and could express their ideas well. This might result in sample bias.

## Conclusions

In this qualitative study, we explored the conceptualization of functioning of people with SMD in a typical rural setting in a low-income country. Participants identified many factors contributing to functional impairment in people with SMD apart from the symptoms associated with the illness. The day- to- day tasks that are valued by the family, neighbors and the community and are crucial for survival were reported to be impaired in people with SMD. Adverse impacts of functional impairment were considered to extend beyond the person with SMD to also affect the functional capacity of the family.

This study indicates that family caregivers have significant impact on the impairment and recovery of people with SMD; and, on the other hand, they are affected by the care burden of their mentally ill family member. Therefore, family members should be involved in the treatment and care of people with SMD where appropriate. In order to bring about functional improvement in people with SMD, there is need to work on improving the treatment being provided as many of the people with SMD were not getting consistent treatment. This can be achieved through giving the care closer to their home, for example, at primary health care center. It would also be important to think about designing appropriate and evidence-based rehabilitation services relevant to the context that may improve the functioning of people with SMD. Tackling social exclusion and poverty is also needed alongside medical treatment. Differences in the functional roles of men and women and the complexity and uniqueness of occupational and social activities in this study setting, support the argument that existing functioning scales are more suited to urban settings, and that there is a need to develop and validate locally appropriate measures of functioning.
